# Unblocking a terminolateral anastomosis on an interposed coloplasty for caustic esophagitis: placement of a lumen-apposing metal stent to straighten the lumen

**DOI:** 10.1055/a-2410-3349

**Published:** 2024-09-19

**Authors:** Mathieu Pioche, Pierre Lafeuille, Alice Burgevin, Marion Schaefer, Olivier Monneuse, Jérôme Rivory, Alexandru Lupu

**Affiliations:** 1 Gastroenterology and Endoscopy Unit, Edouard Herriot Hospital, Hospices Civils de Lyon, Lyon, France; 2 Gastroenterology and Endoscopy Unit, Brabois University Hospital, Nancy, France; 3 Digestive Surgery Unit, Edouard Herriot Hospital, Hospices Civils de Lyon, Lyon, France

The management of caustic esophagitis is complex and is generally carried out in an emergency situation where the prognosis is life threatening.

When esophageal necrosis is major, one of the surgical solutions is esophageal stripping followed by interposition coloplasty with cervical anastomosis 1 . This coloplasty is usually termino-terminal, but sometimes, if the colonic transplant is too long, a termino-lateral anastomosis is chosen to avoid recutting the transplant and shortening it too much.

In the case presented here, a terminolateral anastomosis had to be performed and was complicated by a transcutaneous cervical anastomotic fistula. During examinations with opacification, the contrast product filled the pseudo diverticular aspect of the end of the transplant ( Fig. 1 **a** , Video 1 ) without opacifying the lumen of the underlying coloplasty, with subsequent leakage of the contrast into the cervical fistula.

**Fig. 1:**
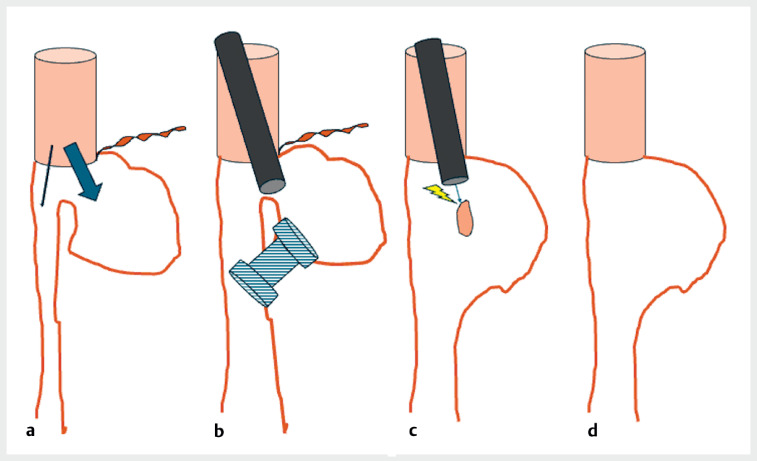
Schematic description of the procedure. **a** Initial aspect with “stricture” of the main lumen of the coloplasty, with diverticular shape of the terminolateral anastomosis with cervical fistula. **b** Placement of a lumen-apposing metal stent between the terminal part of the coloplasty and the main lumen. **c** Mucosal bridge section remaining after removal of the stent. **d** Final aspect after section of the spur.

Terminolateral anastomosis obstruction on an interposed coloplasty for caustic esophagitis: lumen-apposing metal stent anastomosis to straighten the lumen.Video 1

In view of this pseudo diverticulum with spur obstruction, we opted to place a 20-mm lumen-apposing metal stent (LAMS; Axios; Boston Scientific, Marlborough, Massachusetts, USA) between the lateral termination and the lumen of the colonic graft to re-establish a straighter lumen ( Fig. 1 **b** ). This allowed the fistula to dry out immediately after the procedure and the patient could resume liquid feeding.

After removal of the LAMS 3 months later, the fistula had completely dried and we sectioned the residual mucosal spur that had developed around the prosthesis ( Fig. 1 **c** ). On final opacification, esophageal passage was normal with no leakage or obstruction. There was no recurrence of the fistula in the following month and no eating disorders were noted ( Fig. 1 **d** ).

As previously described for strictures 2 , LAMS could be an effective treatment to straighten the way between the lateral pouch and the lumen to remove the obstruction and treat the fistula in terminolateral anastomotic disorders.

Endoscopy_UCTN_Code_TTT_1AO_2AZ

## Notes

Endoscopy E-Videos https://eref.thieme.de/e-videos
*E-Videos* is an open access online section of the journal *Endoscopy* , reporting on interesting cases and new techniques in gastroenterological endoscopy. All papers include a high-quality video and are published with a Creative Commons CC-BY license. Endoscopy E-Videos qualify for HINARI discounts and waivers and eligibility is automatically checked during the submission process. We grant 100% waivers to articles whose corresponding authors are based in Group A countries and 50% waivers to those who are based in Group B countries as classified by Research4Life (see: https://www.research4life.org/access/eligibility/ ). This section has its own submission website at https://mc.manuscriptcentral.com/e-videos .
